# Trade-offs, fairness, and funding for cancer drugs: key findings from a deliberative public engagement event in British Columbia, Canada

**DOI:** 10.1186/s12913-018-3117-7

**Published:** 2018-05-08

**Authors:** Colene Bentley, Sarah Costa, Michael M. Burgess, Dean Regier, Helen McTaggart-Cowan, Stuart J. Peacock

**Affiliations:** 1 Canadian Centre for Applied Research in Cancer Control, BC Cancer, 675 West 10th Avenue, Vancouver, BC V5Z 1L3 Canada; 2 W. Maurice Young Centre for Applied Ethics, School of Population and Public Health, Medical Genetics, Southern Medical Program, University of British Columbia, 1088 Discovery Avenue, Kelowna, BC V1V 1V7 Canada; 3 School of Population and Public Health, University of British Columbia, 2206 East Mall, Vancouver, BC V6T 1Z3 Canada; 4 Faculty of Health Science, Simon Fraser University, Blusson Hall, Room 11300, 8888 University Drive, Burnaby, BC V5A 1S6 Canada

**Keywords:** Public engagement, Cancer drugs, Priority setting, Canada

## Abstract

**Background:**

Spending on cancer drugs has risen dramatically in recent years compared to other areas of health care, due in part to higher prices associated with newly approved drugs and increased demand for these drugs. Addressing this situation requires making difficult trade-offs between cost, harms, and ability to benefit when using public resources, making it important for policy makers to have input from many people affected by the issue, including citizens.

**Methods:**

In September 2014, a deliberative public engagement event was conducted in Vancouver, British Columbia (BC), on the topic of priority setting and costly cancer drugs. The aim of the study was to gain citizens’ input on the topic and have them generate recommendations that could inform cancer drug funding decisions in BC. A market research company was engaged to recruit members of the BC general public to deliberate over two weekends (four days) on how best to allocate resources for expensive cancer treatments. Participants were stratified based on the 2006 census data for BC. Participants were asked to discuss disinvestment, intravenous versus oral chemotherapy delivery, and decision governance. All sessions were audio recorded and transcribed. Transcripts were analyzed using NVivo 11 software.

**Results:**

Twenty-four individuals participated in the event and generated 30 recommendations. Participants accepted the principle of resource scarcity and the need of governments to make difficult trade-offs when allocating health-care resources. They supported the view that cost-benefit thresholds must be set for high-cost drugs. They also expected reasonable health benefits in return for large expenditures, and supported the view that some drugs do not merit funding. Participants also wanted drug funding decisions to be made in a non-partisan and transparent way.

**Conclusion:**

The recommendations from the Vancouver deliberation can provide guidance to policy makers in BC and may be useful in challenging pricing by pharmaceutical companies.

**Electronic supplementary material:**

The online version of this article (10.1186/s12913-018-3117-7) contains supplementary material, which is available to authorized users.

## Background

Expenditure on oncology drugs has risen faster in recent years compared to other areas of health care [1–3]. This is due, in part, to the higher price tags associated with the discovery of new therapies and increased rates of use [1, 4]. While health gains have been made in the treatment of cancer with new therapies [5–7], there is concern among policy makers and health-care providers that the high costs for new cancer treatments might not be justified by the often small increase in health benefit they provide over less expensive drugs [7–10]. In Canada and elsewhere, policy makers are looking for effective ways to allocate drug budgets in the face of climbing prices and increased utilization for oncology drugs, while balancing the need to provide timely and equitable access to innovative treatments [1, 2]. This is focusing attention on the economic and ethical challenges of making coverage decisions that are sustainable and regarded as fair, [11, 12] especially within publicly funded health-care systems like Canada’s [13]. Policy makers must weigh various opportunity costs and determine how best to address dilemmas over sustainability, access, and fairness raised by scarcity and social need.

Increasingly, health policy makers are turning to the public for guidance in developing solutions to policy dilemmas [14, 15]. Incorporating public preferences into health-care decision-making processes can be conducted using priority-setting frameworks such as multi-criteria decision analysis and program budgeting and marginal analysis, which use public opinion in the form of stakeholder input and locally relevant decision-making criteria to support the decision-making process [16, 17]. One-way consultative methods such as focus groups or surveys have traditionally been used to elicit public preferences in these frameworks [18]. However, deliberative forms of public engagement are emerging as a viable policy-informing approach to civic participation in health-care decision making. Specifically, deliberative public engagement involves members of the public in a process of learning and exchanging views explicitly directed towards collective problem-solving, thus making it distinct from other discussion-based consultation forums, like focus groups [14, 19, 20]. As a method, it is highly suited to address topics in health care where some of society’s most ethically, fiscally, and politically challenging decisions are made [14]. In Canada, deliberative engagements have been set up periodically to advise provincial Ministries of Health on decisions related to technology assessment [21–24], health services [25], and policies for biobanks [26].

In cancer control, relatively little is known about what Canadians value—or what values they prioritize—concerning different cancer interventions and their outcomes (Gibson et al, 2013: unpublished manuscript). Canadian studies focused on priority setting in cancer control have typically done so from a clinical or policy maker perspective [27–30]. To address this gap and to support decision makers’ desire for public guidance on oncology treatments [31], we conducted a public deliberation event in September 2014 in Vancouver, British Columbia (BC), on the topic of priority setting and the high cost of cancer drugs. The goal of the Vancouver deliberation was to provide recommendations related to funding decisions for cancer drugs based on the informed and civic-minded deliberations of a socially diverse group of British Columbians. In BC, most prescription drugs listed on the province’s public drug formulary are reimbursed through its PharmaCare program and BC Cancer specifically covers the approved cancer drugs available in BC. To understand participants’ preferences related to drug funding coverage in BC, a well-established approach to deliberative public engagement [26] was adapted and implemented for the project. Titled “Making Decisions about Funding for Cancer Drugs: a Deliberative Public Engagement,” the deliberation brought together 24 members of the general public over two weekends (four days) to discuss how best to allocate resources for costly cancer treatments. Participants discussed disinvestment, intravenous versus oral chemotherapy delivery, and decision governance for funding cancer drugs. Thirty recommendations on disinvestment, cost-benefit trade-offs, and trustworthy governance were generated from the event. A summary of the event and the 30 recommendations is available elsewhere [32]. This paper details the methods of recruitment and deliberation used at the event, and provides in-depth analysis of key recommendations made by participants by contextualizing their recommendations within the dynamics of dialogic exchange amongst all participants during the event. Results are presented from our analysis of the event transcripts, which contain participants’ inter-subjective exchanges over four days. This analysis provides a rigourous understanding of the reasons why participants may have supported—or rejected—a particular recommendation, based on our assessment of their articulations about what was important to them when it comes to funding expensive cancer therapies. The Vancouver study was approved by the University of British Columbia - British Columbia Cancer Agency Research Ethics Board. In 2016, the research team received additional funding from the Canadian Partnership Against Cancer (CPAC) to conduct an additional six public deliberation events, based on the BC model, in communities across Canada to engage Canadians about their priorities for making cancer drug funding fair and sustainable.

## Methods

### Recruitment

The goal of recruitment was to create a “mini public” by bringing together a diversity of perspectives within the BC general public. A mini public is a representation of the larger public from which it is drawn, and thus represents “the diversity of social characteristics and plurality of initial points of view in the larger society” [33]. As a forum for deliberation, a mini public should be free from vested or sectarian interests that can steer discussion in a particular (political) direction and thus undermine civic trust in deliberative processes [34].

A market research company was engaged to oversee recruitment efforts. An online letter of invitation to participate in the deliberation was sent to the company’s panel members. The letter specified the dates and the honorarium for the deliberation ($125/day), invited respondents to complete a brief demographic questionnaire, and informed them they may be selected to complete a preference-based survey (see below). All respondents were stratified by age, sex, geography, ethnicity, income and education, based on the 2006 census data for BC, and screened for experience with chronic disease (y/n), have children (y/n) and residence by BC health authority (there are five BC health authorities). Here, demographic diversity was used as a proxy for experiential diversity. The rationale for screening by chronic disease and parenthood was to include approximately 50% of participants with patient and/or care-giver experience and approximately 50% who have children, since health status and intergenerational relationships provide important perspectives on matters of legacy, inheritance, and sustainability. We also included participants from each of the five BC health authorities, because geography may affect people’s experiences with how health-care is delivered. We oversampled for young males and Aboriginal individuals, since these groups are typically underrepresented in public discourse but contribute to the social pluralism of BC [35]. Individuals were not eligible to participate if they were: health policy makers; employees or those with a direct financial relationship with a tobacco company; individuals who had lobbied for health advocacy groups; unavailable for both event weekends; or had participated in a market research study in the previous six months. A pool of 80 respondents was created at this stage.

The 80 respondents were invited to complete a 16-question preference-based survey that asked them to imagine they had been diagnosed with a serious disease and to select a treatment option based on characteristics such as their health state before and after treatment, pain level, duration of life after treatment, and cost. Respondents were grouped into one of three categories based on their preferences. From this, the research team identified potential participants based on their various life experiences (expressed demographically) and preferences (expressed categorically). Respondents received a $25 honorarium for completing the survey. The preference-based survey was developed specifically for this study and is in Additional file 1.

Using an algorithm developed by the research team, 30 individuals were selected from the 80 respondents and invited to participate in the event. All participants signed a written informed consent form prior to the event. The target sample size of 30 individuals was sufficient for us to stratify individuals for diversity as specified in the recruitment criteria outlined above [35]. The algorithm was also used to find replacements if someone dropped out prior to the event.

### Deliberative public engagement

Deliberative public engagement methods developed by Burgess and O’Doherty [26] were adapted and implemented for this project. The event took place over two weekends and involved small and large group sessions led by trained facilitators. The first weekend was designed to set the foundations for meaningful deliberation by i) providing participants with sufficient and quality information on a range of perspectives related to cancer drug funding (via expert speakers, an information booklet, and a website) and ii) helping people develop the skills required of participants in reasoned, dialogic exchange focused on collective decision making. The purpose of the second weekend was to enable participants to draw on their new skills and knowledge to make recommendations on priority setting in funding for cancer drugs.

Small and large group sessions were also designed to serve different deliberative purposes. The small group sessions were intended to: i) provide a smaller forum to encourage less vocal speakers to participate; ii) model and establish deliberative norms, such as listening with respect, being open to others’ viewpoints, and seeking and providing clarity on positions; and iii) generate a broad range of perspectives on the topic of discussion. Facilitators were to avoid drawing conclusions in the small groups in order to avoid identity formation and the frustration of drawing premature conclusions that may need to be re-justified in the whole group setting. The purpose of the large group deliberation was to i) ensure that participants receive the same information and instructions; ii) introduce various viewpoints aired in the small group sessions to the whole group; and iii) work collectively to formulate recommendations for policy.

The deliberative questions were developed prior to the event and in direct consultation with key decision makers at CPAC, the BC Ministry of Health, BC Cancer, and the pan-Canadian Oncology Drug Review (pCODR). With the exception of the governance question, all deliberative questions were posed first in small groups and then discussed collectively in the large group. Recommendations were made collectively in the large group only.

The recommendations were composed by the participants, who voted on them using electronic clickers. Consensus was not the goal of the deliberations or of the voting exercise. The activity of voting was used by the research team as a tool to determine where participants’ views converged and diverged. Supporting and opposing viewpoints were captured explicitly during the event in order to help decision makers and citizens gain a better understanding of the values they share and don’t share, so they can be incorporated into public policy in a meaningful way. Participants who voted “no” on a recommendation were asked their reasons for disagreeing with it. A participant might disagree because he/she rejected the recommendation, disagreed with its wording, or determined the recommendation was redundant or not necessary. Transparent accountability for participants’ diverse views also supports the trustworthiness and legitimacy of recommendations for decision makers and the wider public.

On the final day of the event (Day 4), participants reviewed and re-voted as a group on recommendations they made the previous weekend. Re-voting gave participants the opportunity to reassess earlier statements and allowed the research team to capture the reasons behind any changed perspectives.

All sessions were audio recorded and transcribed.

#### Transcript analysis

The event format affects the methods used to analyze transcripts. In essence, the data from transcribed proceedings of deliberative public engagements are fundamentally different from the qualitative data generated from interviews or focus groups [36]. Because deliberative events facilitate collectively reached recommendations after a process of learning and engagement with others, deliberants’ knowledge and sense of collectivity accrue over time, as they learn from one another, reflect on what they’ve learned, and then direct their knowledge to collective ends. Interviews and focus groups, on the other hand, elicit different types of knowledge over much shorter time spans, with interviews being repeatable instances of the same phenomena (i.e., different subjects answering a set roster of questions). Deliberative proceedings thus are not amenable to straightforward thematic or content analysis, since participants’ statements on Day 1 and Day 4 cannot be weighted equally, and more significance must be accorded to statements that are more informed and more collectively-oriented than other statements [36]. Moreover, because of the dialogic and reason-giving nature of deliberation, participants may change their minds over time in response to increased knowledge or more persuasive arguments. Changeability under these conditions is positively valued; it is not regarded as contradictory or incoherent, as it may be if all statements were weighted equally.

The transcripts were entered into NVivo qualitative data analysis software (QSR International Pty Ltd., Version 10, 2012) for coding. Both deductive and inductive coding were used. Deductive coding begins with established categories and codes, which are developed from the scholarly literature and research experience. Here, the deliberative questions and the layout of the event (e.g., small and large group settings, Day 1, Day 2, etc.) helped set the categories in advance of the deliberation itself. Inevitably, coding deductively imposes constraints on what discursive content is captured and thus available for analysis. To combat these constraints, inductive (or “open”) coding methods were used to capture emergent ideas. Participants were de-identified and given a number (e.g., Participant 1, Participant 2, etc.) during transcript analysis.

All sessions were coded and analyzed by one reviewer and a second reviewer independently coded 37% of the transcripts. An average Kappa score of 0.67 was achieved between the reviewers for all nodes and sources (unweighted). Weekly norming sessions were also held to review, track, and resolve issues as they arose.

## Results

A total of 24 individuals participated in the deliberation. Participants’ characteristics are in Table 1. Chi-square tests showed that the final 24 participants met our recruitment criteria. Participants made a total of 30 recommendations over the four days. The full list of recommendations is reported elsewhere [32]. Below is an analysis of key recommendations from the Vancouver event. The terms “Most” and “All” beside each recommendation indicates whether or not the recommendation had consensus, even though consensus was not the goal of the event or of making recommendations.

**Table 1:** Participants’ characteristics

Characteristics	Count N (%)
Sex	
Female	13 (54.2%)
Male	11 (45.8%)
Age (years)	
18–24	1 (4.2%)
25–34	5 (20.8%)
35–49	4 (16.7%)
50–64	9 (37.5%)
65+	5 (20.8%)
Residence by Provincial Health Authority^a^	
Fraser	7 (29.2%)
Vancouver	8 (33.3%)
Interior	4 (16.7%)
Island	4 (16.7%)
Northern	1 (4.2%)
Experience with chronic illness (personal or as caregiver)	
No	16 (66.7%)
Yes	8 (33.3%)
Ethnicity	
Caucasian	16 (66.7%)
Chinese	3 (12.5%)
South Asian	1 (4.2%)
Aboriginal	1 (4.2%)
Other	3 (12.5%)
Highest education level attained	
High school	6 (25.0%)
Some university	2 (8.3%)
University or College	16 (66.7%)
Annual household income	
Less than $20,000	3 (12.5%)
$20,000 - $34,999	3 (12.5%)
$35,000 - $49,999	3 (12.5%)
$50,000 - $79,999	7 (29.2%)
$80,000 or above	8 (33.3%)
Have children?	
Yes	13 (54.2%)
No	11 (45.8%)

^a^A map of the Health Authorities in BC can be found here: https://www2.gov.bc.ca/gov/content/health/about-bc-s-health-care-system/partners/health-authorities/regional-health-authorities

### Deliberative question on disinvestment: Under what circumstances is there an obligation to continue to fund a cancer drug when new information suggests the drug is not as desirable as previously determined?

Recommendations:

- There is an obligation to continue to fund a cancer drug if discontinued funding would have a negative impact on populations in rural communities and others with limited access. (All)
- There is an obligation to continue to fund a cancer drug if it is significantly easier to use compared to other drugs or treatments (for example, oral vs. intravenous drugs). (Most)

In their deliberations about disinvestment, participants voiced concerns over equity of access and patients’ ability to tolerate an alternative treatment in the event that a drug is removed from the public drug formulary (a public drug formulary is a list of drugs and drug products covered by a province’s publicly-funded health-care plan). Their concerns over equity of access were expressed in terms of how delisting a drug would affect those in “rural communities and others with limited access”. Participants felt it was unacceptable to delist a drug if doing so created barriers to health-care utilization for patients living in rural and remote areas, particularly if the drug being delisted was an oral medication that could be taken at home instead of an intravenous (IV) drug that may require patients to travel long distances to receive. They also worried that disinvestment decisions might create have and have-not patients:

Participant 4: You have to keep the funding going for these people [already on the drug] because you can’t just [say]…there’s a new one for us city dwellers or whatever. So I think …if you’re going to stop [funding it, then] stop it…The new drug is available to all people. You can’t split it up.

(Small group G, Day 2)

“Split[ting]” the drug between urban and rural patients would result in discriminatory practices that were unacceptable to participants. They suggested instead that the obligation to fund a drug extended beyond rurality to include patients “with limited access,” such as patients with mental illness, and those who were homeless, housebound, First Nations, or had mobility constraints. One participant summarized the discussion from her small group, saying, “We were really concerned about fairness around the availability of drugs” (Participant 1, Large group, Day 2).

Concerns over access and fairness when delisting a drug were also evident in participants’ lengthy discussion of what they meant by “significantly easier to use” in their second recommendation. For several deliberants, “easier to use” referred to a preference for oral over IV drugs. One participant described IV drugs as “very hard on your veins…and corrosive to your body” and would take a pill over IV chemotherapy “any day” (Participant 18, Large group, Day 3). Some participants wanted a more expansive definition of “easier to use” to include patients’ ability to tolerate the replacement drug, independent of whether the replacement drug is more convenient to use:

Participant 1: We are also saying that, in addition to people who might live in rural areas, maybe somebody is unable to take a particular pill that needs to be taken with a glass of milk because they can't drink milk, and this new drug can only be taken with milk. So now all of a sudden the people who are allergic to milk cannot take this new drug, because it is not as easy to use as the old one.

(Large group, Day 2)

Once again participants worried that disinvestment decisions would create opportunities to discriminate between categories of patients. Although taking a pill for chemotherapy may be preferable to IV treatment for many patients, it may not be the case for patients who cannot tolerate the oral treatment.

Disinvestment and fairness were also linked to patients’ choice. Participants argued that patients should be able to remain on a drug that works for them, even if a policy decision is made to delist the drug:

Participant 4*:* So I think the patient should have some say in….may decide, for whatever reason, she would prefer the older [drug].

Participant 18*:* Yeah, I had to take Tamoxifen for five years….I had to stop taking it because it was just making my life hell. So they gave me Zoladex which…didn’t give me the same side effects. So I think, yeah, like I agree with that.

(Small group G, Day 2)

Participant 6: I think it’s just, like, when you’re sick and stuff like that, and you’re on a certain drug, that being switched from one drug to another drug [---] is just disturbing.

(Small group Y, Day 3)

As these discussions show, patient choice was less about autonomy and more about having the chance for a better quality of life. This point was expressed emphatically in Weekend 2’s recommendation on disinvestment: “Patients who are taking an existing drug should have the option to stay on the existing drug even if it is more expensive than a similar new drug.” This recommendation was nicknamed “the grandfather clause.” It showed that opportunities for fairness and a better quality of life for patients who are stabilized on a current treatment were important enough to participants that they were unwilling to trade these opportunities to realize cost savings when budgets are limited.

### Decision scenarios 1 and 2: Trade-offs between cost and quality of life or length of life

**Fig. 1:**
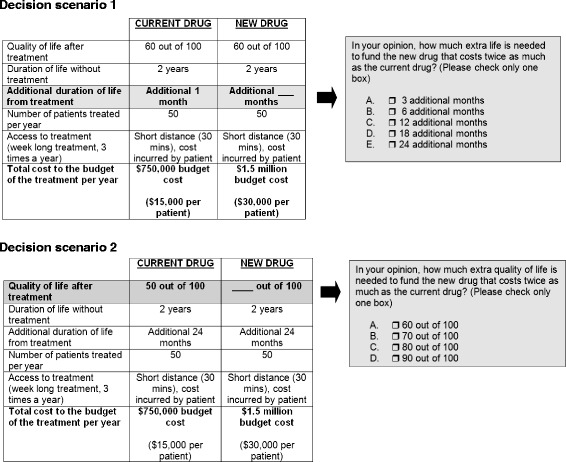
Decision scenarios. Participants were given the following instructions and decision scenarios on Day 3 of the deliberation: Imagine you are decision-makers, responsible for the cancer budget. The decision you make will affect the people in your province only. A fixed budget has been set aside to fund one treatment. The budget is not large enough to fund both of the treatments. The budget will only fund healthcare and cannot be used for research that may improve a patient’s condition in the future. The total cost of the treatment includes all costs that are relevant to the decision. (Decision scenario 1): Trade-off between cost and additional length of life. (Decision scenario 2): Trade-off between cost and increased quality of life

**Fig. 2:**
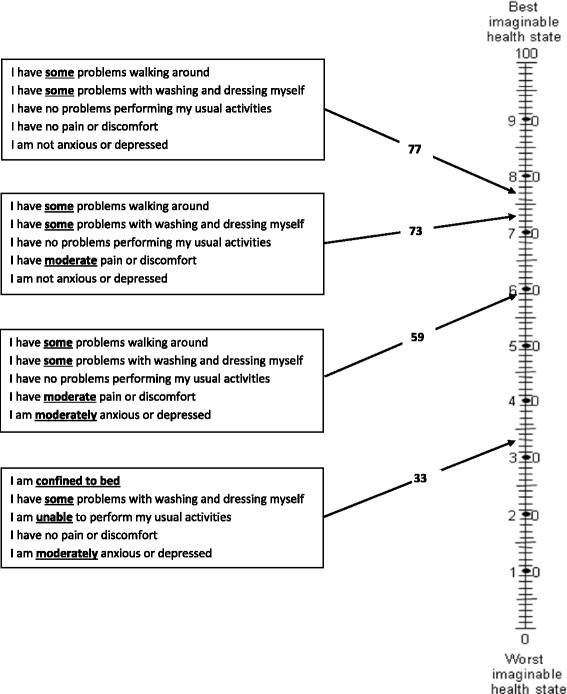
Quality of Life Scale. Note: Usual activities means things like work, study, family, and leisure activities

Four decision scenarios were presented to participants and administered on the second weekend of the event (Day 3). The two decision scenarios are shown in Fig. 1. They were introduced because the research team felt participants were ready to make explicit trade-offs. The Quality of Life (QoL) scale (Fig. 2) was also explained, including the scale’s use in health research and its subjective nature. They were told that most people on average place themselves at 80–90 on the scale. Participants were also given quality of life scores for Canadians (Table 2). Participants then broke into small groups to discuss each scenario.

**Table 2:** Quality of Life Scores for Canadians. Participants were given the following information about the age groups and average quality of life scores for Canadians (men and women)

Age group	Average score
18–29	83
30–39	83
40–49	81
50–59	81
60–69	78
70–79	73
80+	65

The scenarios are constructed situations: participants were asked to play the role of decision makers with fixed budgets who must make a decision between two treatment options. The variables within the scenarios were held constant to force the trade-off and the specific trade-off options—in the form of additional months of life or extra quality of life—were also constrained. Participants’ reasons for selecting a particular trade-off are more important than the numeric trade-off selected.

Two scenarios are discussed, below.

### Decision scenario 1: In your opinion, how much extra length of life is needed to fund the new drug that costs twice as much as the current drug?

Recommendation:

- There needs to be a minimum of 12 months of additional duration of life. (Most)

Participants’ approach to this scenario varied. Some made mathematical calculations on how much extra life is needed to justify twice the cost. Others felt that because 60 on the QoL scale was not a good health state—and because patients’ health state in the scenario did not improve with the new drug—they wanted greater duration of life for the money. Several participants wanted a “significant improvement” in the amount of additional life to justify twice the cost:

Participant 11*:* I will say the one thing I have noticed as a group, none of us ha[s] picked the minimum option.

Participant 10: Yeah.

Participant 11: We’ve all expected a little bit more.

Participant 17: Yeah, significant, yes.

Participant 11: -- significant improvement if we’re going to spend twice as much

(Small group B, Day 3)

Participant 1: I picked 12 [months].

Facilitator: 12 [months]. Is there a reason for that?

Participant 1: Because three months and six months is not a very long time, and especially if you’re looking at a 60 percent quality of life….[P]art of that’s going to be taken up with, like, being sick, or having to have treatment. And really, like, three to six months…hardly seems worth it for double the cost.

(Small group Y, Day 3)

Participants considered “double the cost” to be a large expenditure and thus wanted more than the minimum amount of additional months of life from the new drug. Concern for a better return on investment pushed participants to question the worthwhileness of investing “significant” amounts of money in a new drug that showed only a small increment in health benefit. Getting good value for money was also important in the large group discussion of this scenario:

Participant 13: [S]ix to twelve [months means] you got time to get your stuff together, and approach [the end of life]….[F]or some people six months is plenty, for other people a year.

Participant 22: I think the 12 months and higher was if you looked at only money....I sat there trying to decide between 12 and 6 [months] because I’m like, well, if we’re talking dollars, 12. If we’re talking emotions, 6.

(Large group, Day 3)

This quotation shows participants’ desire to be good stewards of public finances (“I think the 12 months and higher was if you looked at only money”). It also demonstrates their effort to strike a balance between civic responsibility and compassion for others (“[I]f we’re talking dollars, 12. If we’re talking emotions, 6”), and balance collective with personal viewpoints. Decision makers endeavour to strike this difficult balance all the time. Having sufficient time to “get your stuff together” or complete a ‘bucket list’ were additional reasons for supporting more than minimum amounts of extended life for double the budget. Several felt that it was unrealistic to expect 18–24 months of extra life from the new drug.

Not all participants supported the recommendation. Some were willing to pay twice the price for a new drug that extended patients’ lives by fewer than 12 months:

Participant 3: I [was] saying to my group, I was like, the last week of mom's life, for five more minutes I’d have given like $10,000. So, for me it's really hard to just put that dollar amount on that amount of time. So I'm like six months, I would take it.

Participant 24: Six for me as well.

Participant 7: I would say three to six....Every moment is precious and time matters.

(Large group, Day 3)

Here, participants resisted trading value for money when the benchmark for value was set at 12 months by the group. Unsatisfied with the 12-month threshold, they considered fewer months of extended life to be acceptable because “every moment is precious” even when drug costs are high and budgets are limited.

Participants’ quantitative trade-offs—i.e., whether they were willing to trade 3, 6, or 12 months of additional life at a “significant” cost—are less important than their reasons for selecting them. For this scenario, participants accepted that trade-offs are necessary and most wanted to see more than a minimum increase in health benefit from expensive drugs. Participant 7 was the only participant to consider the minimum health benefit (i.e., 3 months) as a suitable trade-off for a drug that costs twice as much.

### Decision scenario 2: In your opinion, how much extra quality of life is needed to fund the new drug that costs twice as much as the current drug?

Recommendation:

- There needs to be a minimum of 20 points of improvement in quality of life. (Most)

Overall, participants described a meaningful quality of life as being able to think and care for oneself, spending time with loved ones, and living without pain or nausea; for some it meant being able to work. They related their descriptions to the QoL scale and the decision scenario. As expected, the points on the scale meant different things to different people. For instance, one person interpreted 60 on the scale as being bedridden and therefore undesirable. Some interpreted 50 on the scale as a manageable health state, whereas others considered 50 to be a poor health state. Most felt that 70 on the scale meant you were still a productive member of society:

Participant 3: I was just going to say, like, for a specific percentage… our entire group also said 70. Because I feel like, 80 to 90 is really good. Like generally what we all feel now. And so I feel like you could...justify doubling the cost feeling a little bit crappier than you do now, but you don't really want to go down too far. So we all ended up picking 70.

Facilitator: 70?

Voices: Yeah. Yes.

(Large group, Day 3)

Participants wanted to see a clear improvement in quality of life from the new drug if it were to cost twice as much as the current treatment. They quantified the improvement as an increase of 20 points on the QoL scale—that is, a climb from 50 to 70 points—yet determined that 80 or 90 on the scale was, as one participant put it, “optimum health, and maybe not that achievable” for a cancer patient (Participant 23, Large group, Day 3). One small group also wanted to see “marked improvements” in health benefit from the new drug in order to send a message to pharmaceutical companies: “By having [quality of life] at a minimum of 70, what it does is it also helps to promote the idea that if you want us to fund your drugs at double the cost, we want to see marked improvements” (Participant 14, Small group R, Day 3).

Participants who disagreed with the recommendation were largely unpersuaded by value for money assessments when it came to patients’ quality of life. They considered 10 points’ increase on the scale to be an acceptable magnitude of benefit despite the expense:

Participant 4: I have cancer...and I'm not expected to get 70 or 80 [on the QoL scale]…. So if I could get 10 percent, that is a lot in a cancer patient….Ten percent quality improvement is -- I could live with that.

Participant 7: To me 10 percent is good. It makes a big difference, and I totally agree with what you're saying. I worry that we're -- when we say things like 20 [points on the QoL scale] and again, with the 12 months [additional length of life] too, we're setting the bar too high.

(Large group, Day 3)

Those who disagreed with the recommendation felt they could justify—i.e., “could live with”—a decision to spend twice as much on a new drug in return for small gains in patients’ quality of life. Quality of life was clearly important to all participants, with some willing to lower the threshold of benefit from expensive drugs in order to get it.

### Deliberative question on governance: What would make drug funding decisions trustworthy?

Recommendations:

- There is a need for transparency around how drug funding decisions are made, what stakeholders are involved, and possible conflicts of interest. (All)
- There is a need for an independent body that would oversee and review drug funding decisions and involve a variety of people without political motivations (participants were concerned about patronage). (Most)

The deliberative question on trustworthy governance was discussed on the final day of the event and in the large group only. Above all, participants wanted reassurances that drug funding decisions were made in a non-partisan and transparent way. They wanted members of drug funding committees to be health-care professionals like oncologists and pharmacists, and to include patients and members of the public. Committee members would be “hired, not appointed” (Participant 2, Large group, Day 4) to prevent pharmaceutical companies and political appointees from influencing decisions and to keep the process free of nepotism. In addition, the public would have confidence in decisions reached by the committee if its decision-making process and outcomes were placed in the public domain:

Participant 18: I'm going to, you know, trust how they make decisions on drug funding [if I know how] they make their decision….[I]t would be nice to know how they make their decision in a broad way…and publish that to the public as well.

(Large group, Day 4)

Participants agreed that posting this information on a website, along with clinical trials data and input from patients and the public, would enhance people’s trust in decisions about funding for cancer drugs. The BC government and the Canadian Agency for Drugs and Technologies in Health (CADTH) publish their drug reviews and health technology assessments (HTAs) on their websites. Even after learning this was the case, participants wanted to express their strong preference for transparency and trustworthiness in HTA-type decision processes by making a recommendation to this effect.

Participants also agreed on the need for increased oversight of drug funding decisions. They wanted a committee or “independent body” to have the authority to review drug funding decisions on a regular basis. Its mandate would be to:

Participant 13*:* Oversee and review.

Participant 11: [O]versee kind of means they have the right to kind of step in and change things….[I]f they are just reviewing it and looking for conflicts then they can point those out.

(Large group, Day 4)

Participants believed it was important to have in place a mechanism or opportunity to revisit and revise current decisions (i.e., to “step in and change things”) when necessary.

## Discussion

Deliberative engagement events are opportunities to garner informed public input on pressing policy questions. The Vancouver deliberation brought together 24 British Columbians with various demographic and preference characteristics so that a range of experiences and perspectives could inform the consultation on setting priorities for cancer drug funding in BC. In addition, the structure of the deliberation—for instance, small and large group discussions over two weekends, supporting deliberation with information from expert speakers and a booklet, and using decision tools like the scenarios and a voting system—involved participants in a process of discussion and reflection directed at finding collective solutions to funding high-cost cancer drugs when resources are limited.

Significantly, participants accepted the premise that budgets are limited: no one said “fund everything.” They understood the necessity of making cost-benefit assessments as a way of addressing complex social problems, like health care. This was evident in their discussion of the decision scenarios, when they were asked to assume the role of decision makers. Faced with a fixed budget and a new treatment that cost twice as much as the current treatment, participants-as-decision-makers by and large wanted a “marked improvement” in health benefit given the budget impact. While not all participants agreed on 6 or 12 months of additional length of life or 20 points improvement on the QoL scale for double the cost, they nonetheless supported the principle of getting good value for money—or a good return on investment—when setting health policy. They accepted cost as a unit of evaluation across contexts and treatments.

Participants also accepted using cost to set thresholds. It is worth recalling that the thresholds participants set were hypothetical, like the scenarios themselves. For instance, it may be unrealistic to expect a magnitude of 12 months of additional life from today’s oncology drugs, as many participants did, given the small gains in effectiveness seen from some new cancer drugs [7–10]. It is also unrealistic to expect quantitative or generalizable thresholds from deliberative events that can be applied to other decisions. Numeric standards for trade-offs are only possible using quantitative methods either to aggregate across disagreements or derive the majority perspective. However, both of these approaches are inconsistent with qualitative research generally and, in this instance, with the goals of deliberative public engagement, which is to understand and respect the ways in which an informed public comes to agree or disagree over what the most important consequences are of a given funding decision.

In setting thresholds, participants told us what was important to them independent of the numeric value of the trade-off. For example, quality of life was more important to them than quantity of life, since they were willing to accept smaller increases in quality of life from a high-cost cancer drug but demanded greater gains in length of life to justify the same expenditure. In other words, they wanted to see greater than minimum increments in effectiveness from some high-cost drugs—like those that extend life—when they rejected small health gains as insufficient for the price. Understanding the public’s appetite for types of trade-offs provides valuable guidance to decision makers when weighing the opportunity costs related to fair and sustainable funding decisions.

Cost was not the sole factor in setting thresholds, however. It was cost and a capacity to consider others’ perspectives when forming group recommendations that pushed participants to set thresholds at greater than minimum values. Participants demonstrated this capacity for civic-mindedness when they strove to balance personal and social perspectives when setting thresholds. For instance, some were concerned about compassion for others’ pain (“10 percent improvement [on the QoL scale] is a lot in a cancer patient”) if the cost-benefit threshold is set too high. Others reflected on the challenge of balancing responsibility for public resources with the impact of policy decisions on individual lives (“If we’re talking dollars, 12 [months]. If we’re talking emotions, 6”). Significantly, participants who self-identified as patients also took up the challenge the scenarios presented, which was to adopt the (relatively disinterested) perspective of a decision maker in resolving policy dilemmas, as this quotation from a participant-patient shows:

Participant 4: I think you're right. For myself personally, 60 [on the QoL Scale] I can live with. But if I'm looking at the cost, which this whole thing is about, I think you're right: 70 cost-wise makes more sense. You know, if I…took the cost out, I would live with 60. I would take anything. But cost-wise, to make [the decision] relevant and beneficial for more people, you know, you have to [look at it] that way.

(Small group G, Day 3)

Arguably, disinterestedness is simply an abstraction, and thus an unattainable ideal for decision makers and citizens alike. Moreover, we cannot know for certain what all participants—including participant-patients—had in mind when they voted in favour of a particular recommendation. Yet we do know that participants entertained alternative perspectives when they spoke of trying to justify cost-benefit trade-offs that are “relevant and beneficial for more people” alongside personal wants (“I would take anything”) or, again, when they tried to balance costs with compassion. These instances support previous research that shows the public and patients are not too self-interested to participate meaningfully in policy-type discussions [37] or that patients and the public do not necessarily have irreconcilable perspectives on health care. They also offer decision makers additional confidence that the recommendations were collectively reached.

Participants’ more concrete recommendations related to trustworthy governance. Decision makers can derive useful guidance from the participants’ call for drug funding decisions to be made in a transparent and non-partisan way, and the need for independent governance and review of oncology drug funding decisions in BC. Members of the public, patients, and other health-care professionals would compose the governing body. Under these conditions, participants are likely to trust drug funding decision processes and results. Although the BC government and CADTH publish drug reviews on their websites, neither BC nor Canada (at the national level) currently has a mechanism that provides this kind of oversight on an ongoing basis. The practicalities of implementing such a body may not be concrete—participants were not tasked with implementation considerations because they do not have the relevant expertise—yet the recommendations can nonetheless guide practical choices about building infrastructure to identify and support publically acceptable trade-offs for costly cancer drugs.

A limitation of this study is that the results cannot be extrapolated to other contexts. The event and its participants were socially situated, meaning the event constituted a specific collection of people deliberating under specific conditions at a specific time. For this reason, the recommendations cannot be generalized to other publics and locations. Of course, the goal was not to produce an account of how the population of BC would draw on their specific experience to express their individual preferences. However, precisely because the recommendations are the result of informed and reflective deliberation—that is to say, they were made in the context of considering alternative perspectives and reasons for them—the principles underpinning the recommendations are likely to be more durable because they were arrived at from multiple perspectives and account for multiple forms of reasoning.

A second limitation of the study also relates to generalizability in the use of decision scenarios. Constraints inherent in decision scenarios means any recommendations produced from them will be similarly constrained. In this study, the recommendations produced from the scenarios were constrained because: i) the scenarios inevitably focused on some decision assumptions and excluded others (e.g., stage of disease, treatment type, overall budget), which limited the trade-offs considered; and ii) the numeric values of certain recommendations (e.g., “There needs to be a minimum of 12 months of additional duration of life”) are artificial and must not be interpreted at face value. In addition, the use of tools like decision scenarios means that other implications of funding for cancer drugs—for instance, funding for rare cancers—were not explored in relation to issues of fairness and sustainability. Throughout the event, the researchers sought a balance between providing enough relevant information to support quality deliberation and avoiding the provision of too much or certain types of information that would shift the focus away from the public policies the deliberations were meant to inform, as per the pre-event consultations with decision makers.

## Conclusion

The Vancouver deliberative public engagement event and participants’ recommendations provide a set of baseline perspectives on what participants collectively thought made for good, trustworthy decisions about funding for cancer drugs in BC. Analysis of the event transcripts showed that participants grasped the core issues under consideration, and identified cost-benefit and equity trade-offs through respectful and civic-minded deliberation, thereby demonstrating the public can be objective and participate meaningfully in policy-type discussions. Participants accepted the principle of resource scarcity and the consequent need of governments to make difficult trade-offs when allocating health-care resources across the population. When costs are high, particularly in the area of oncology drugs, they supported the view that cost-benefit thresholds must be set. As such, they expected reasonable health benefits in return for large expenditures, and supported the view that some drugs do not merit funding. Participants also called for drug funding decisions to be made in a non-partisan and transparent way. Together, the recommendations arising from the Vancouver deliberation can provide guidance to policy makers in BC and may be useful to challenge pricing by pharmaceutical companies.

## Additional file

Additional file 1: Preference-based Survey. Potential participants completed this 16-question preference-based survey of their preferences for different types of treatments as part of the recruitment strategy for the deliberation. (DOC 181 kb)

## Abbreviations

BC: British Columbia
CADTH: The Canadian Agency for Drugs and Technology in Health
CPAC: The Canadian Partnership Against Cancer
HTA: Health technology assessment
IV: Intravenous
pCODR: The pan-Canadian Oncology Drug Review
QoL: Quality of life
