# Supplementary material for: Trade-offs, fairness, and funding for cancer drugs: key findings from a deliberative public engagement event in British Columbia, Canada
Source: BMC Health Serv Res. 2018 May 8;18:339. doi: 10.1186/s12913-018-3117-7 (PMC5941483; doi:10.1186/s12913-018-3117-7)
Supplement: Supplementary file 1 — Preference-based Survey. Potential participants completed this 16-question preference-based survey of their preferences for different types of treatments as part of the recruitment strategy for the deliberation. (DOC 181 kb) [file 12913_2018_3117_MOESM1_ESM.doc]

**Additional file 1: Preference-based Survey**

***Understanding your preferences for treatment of serious diseases***

Researchers at the University of British Columbia and British Columbia Cancer Agency are asking for your help with a research study. We want to understand what you think about different types of treatments. To do this, we would like you to imagine that you have been recently diagnosed with a serious disease. In the following questions, we will describe how your health will be affected by the disease. We then ask you to answer several questions about different treatment options for your disease that may be available to you.

If you have any questions or concerns regarding this survey, please contact the lead researcher, Dr. Dean Regier at (604)675-8000 ext: 7079, email dregier@bccrc.ca. This research is funded through a grant by the Canadian Institutes for Health Research and Michael Smith Foundation for Health Research.

Please print your name here:

________________________________

Thank you for your time and assistance,

Dean Regier

[dregier@bccrc.ca](mailto:dregier@bccrc.ca)

604-675-8000 extension 7079.

1INTRODUCTION: YOUR HEALTH

**Imagine that you are experiencing changes to your health.** This has prompted you to get examined by your doctor. Your doctor has given you a choice between 3 treatments. The treatment choice differs depending on the following factors.

**1. Your health after you have received treatment**

The treatment will affect your health. Imagine your health after treatment is affected by two factors:

1. Your ability to perform usual activities (e.g., work, study, housework, family or leisure activities)
2. The intensity of pain you experience ( no pain, moderate pain, extreme pain)

|  |  |  |  |  |  |  |  |  |  |
| --- | --- | --- | --- | --- | --- | --- | --- | --- | --- |
| 1 | 2 | 3 | 4 | 5 | 6 | 7 | 8 | 9 | 10 |
| None | |  |  | Moderate | |  |  | Extreme | |

**2. Duration of life after treatment**

The treatment not only can change your health but may also affect how long you can live for.

1. For example, after the treatment ,you may live for an additional 1 year
2. With another treatment, you will live an additional 10 years

**3. Treatment Cost**

In Canada, treatment is paid for by the government. However, you pay for health care indirectly through taxes. Imagine the available treatments for your condition are new. Also imagine everybody in British Columbia (including you) will need to pay for the new treatment through increased taxes on personal income. The increase in personal income tax will be for 1 year only.

- - For example, the total cost that you will need to pay in personal income tax for the treatment is **$250.** This tax payment will be a one-time increase to your personal income tax.
  - Another treatment can cost you a total of **$5000.** This tax payment will be added to your income tax. It will be a one-time increase to your income tax.

Understanding your preferences for treatment

Before you tell us which treatment you prefer, we want to ask you to help us with a problem we have in studies like this one.

In Canada, health care is paid for by the government. Patients and families are rarely are told about the costs of care. In the following exercises, we ask you to consider costs of treatment. When completing each exercise, it may be easier to just notice that one treatment costs more than another.

For example, suppose the cost levels are $100, $200, $500, and $900. People can think of them as “very low”, “low”, “medium”, and “high”. They don’t really think about what they would have to give up out of their monthly budget – such as restaurant meals or new clothing – to pay for the treatment.

Please help us value treatment accurately by paying attention to the actual costs before deciding which treatment options you prefer.

2WHICH TREATMENT DO YOU PREFER**?**

**THIS IS AN EXAMPLE QUESTION. Which treatment do you prefer?** You must choose one treatment option. Each question first describes your health today. A treatment is then offered. The person who answered the question below decided that Treatment B was better than Treatment A or Treatment C. They made this decision by comparing the characteristics of each treatment. There were also fairly certain of the choice they provided.

**Your health today**

**Usual activities:** You have some problems performing usual activities

**Pain:** You are experiencing moderate pain or discomfort

**Duration of life without treatment:** 2 months

|  | **Treatment A** | **Treatment B** | **Treatment C** |
| --- | --- | --- | --- |
| Your health after treatment | Usual activities: some problems  Pain: extreme | Usual activities: some problems  Pain: none | Usual activities: some problems  Pain: moderate |
| Duration of life  after treatment | 4 years | 10 years | 10 years |
| One-time Extra tax payment | $2,700 | $8,100 | $900 |

Which treatment do you prefer? Treatment A Treatment B Treatment C

(please check one box) ❒ **🗹** ❒

How certain were you of your choice?

| 1 | 2 | 3 | 4 | 5 | 6 | 7 |
| --- | --- | --- | --- | --- | --- | --- |
| Guessing at my choice |  |  | Fairly certain of my choice |  |  | Certain I made the right choice |

**Question 1: Which treatment option do you prefer?**

| **Your health today**  **Usual activities: You have some problems performing usual activities**  **Pain: You are experiencing moderate pain or discomfort**  **Duration of life without treatment: 2 months** |
| --- |

|  | **Treatment A** | **Treatment B** | **Treatment C** |
| --- | --- | --- | --- |
| Your health after treatment | Usual activities: some problems  Pain: extreme | Usual activities: some problems  Pain: none | Usual activities: some problems  Pain: moderate |
| Duration of life  after treatment | 1 year | 4 years | 10 years |
| One-time Extra tax payment | $2,700 | $8,100 | $900 |

Which treatment do you prefer? Treatment A Treatment B Treatment C

(please check one box) ❒ ❒ ❒

How certain were you of your choice?

| 1 | 2 | 3 | 4 | 5 | 6 | 7 |
| --- | --- | --- | --- | --- | --- | --- |
| Guessing at my choice |  |  | Fairly certain of my choice |  |  | Certain I made the right choice |

**Question 2: Which treatment option do you prefer?**

| **Your health today**  **Usual activities: You have some problems performing usual activities**  **Pain: You are experiencing moderate pain or discomfort**  **Duration of life without treatment: 2 months** |
| --- |

|  | **Treatment A** | **Treatment B** | **Treatment C** |
| --- | --- | --- | --- |
| Your health after treatment | Usual activities: some problems  Pain: moderate | Usual activities: some problems  Pain: none | Usual activities: no problems  Pain: none |
| Duration of life (years) after treatment | 4 years | 10 years | 1 year |
| One-time Extra tax payment | $450 | $2,700 | $900 |

Which treatment do you prefer? Treatment A Treatment B Treatment C

(please check one box) ❒ ❒ ❒

How certain were you of your choice?

| 1 | 2 | 3 | 4 | 5 | 6 | 7 |
| --- | --- | --- | --- | --- | --- | --- |
| Guessing at my choice |  |  | Fairly certain of my choice |  |  | Certain I made the right choice |

**Question 3: Which treatment option do you prefer?**

| **Your health today**  **Usual activities: You have some problems performing usual activities**  **Pain: You are experiencing moderate pain or discomfort**  **Duration of life without treatment: 2 months** |
| --- |

|  | **Treatment A** | **Treatment B** | **Treatment C** |
| --- | --- | --- | --- |
| Your health after treatment | Usual activities: some problems  Pain: moderate | Usual activities: some problems  Pain: extreme | Usual activities: no problems  Pain: none |
| Duration of life (years) after treatment | 10 years | 6 months | 4 years |
| One-time Extra tax payment | $900 | $8,100 | $2,700 |

Which treatment do you prefer? Treatment A Treatment B Treatment C

(please check one box) ❒ ❒ ❒

How certain were you of your choice?

| 1 | 2 | 3 | 4 | 5 | 6 | 7 |
| --- | --- | --- | --- | --- | --- | --- |
| Guessing at my choice |  |  | Fairly certain of my choice |  |  | Certain I made the right choice |

**Question 4: Which treatment option do you prefer?**

| **Your health today**  **Usual activities: You have some problems performing usual activities**  **Pain: You are experiencing moderate pain or discomfort**  **Duration of life without treatment: 2 months** |
| --- |

|  | **Treatment A** | **Treatment B** | **Treatment C** |
| --- | --- | --- | --- |
| Your health after treatment | Usual activities: some problems  Pain: none | Usual activities: some problems  Pain: moderate | Usual activities: some problems  Pain: extreme |
| Duration of life (years) after treatment | 1 year | 10 years | 6 months |
| One-time Extra tax payment | $450 | $900 | $8,100 |

Which treatment do you prefer? Treatment A Treatment B Treatment C

(please check one box) ❒ ❒ ❒

How certain were you of your choice?

| 1 | 2 | 3 | 4 | 5 | 6 | 7 |
| --- | --- | --- | --- | --- | --- | --- |
| Guessing at my choice |  |  | Fairly certain of my choice |  |  | Certain I made the right choice |

**Question 5: Which treatment option do you prefer?**

| **Your health today**  **Usual activities: You have some problems performing usual activities**  **Pain: You are experiencing moderate pain or discomfort**  **Duration of life without treatment: 2 months** |
| --- |

|  | **Treatment A** | **Treatment B** | **Treatment C** |
| --- | --- | --- | --- |
| Your health after treatment | Usual activities: some problems  Pain: extreme | Usual activities: some problems  Pain: moderate | Usual activities: no problems  Pain: none |
| Duration of life (years) after treatment | 4 years | 1 year | 6 months |
| One-time Extra tax payment | $900 | $8,100 | $450 |

Which treatment do you prefer? Treatment A Treatment B Treatment C

(please check one box) ❒ ❒ ❒

How certain were you of your choice?

| 1 | 2 | 3 | 4 | 5 | 6 | 7 |
| --- | --- | --- | --- | --- | --- | --- |
| Guessing at my choice |  |  | Fairly certain of my choice |  |  | Certain I made the right choice |

**Question 6: Which treatment option do you prefer?**

| **Your health today**  **Usual activities: You have some problems performing usual activities**  **Pain: You are experiencing moderate pain or discomfort**  **Duration of life without treatment: 2 months** |
| --- |

|  | **Treatment A** | **Treatment B** | **Treatment C** |
| --- | --- | --- | --- |
| Your health after treatment | Usual activities:  no problems  Pain: none | Usual activities: some problems  Pain: none | Usual activities: some problems  Pain: extreme |
| Duration of life (years) after treatment | 4 years | 1 year | 6 months |
| One-time Extra tax payment | $2,700 | $450 | $8,100 |

Which treatment do you prefer? Treatment A Treatment B Treatment C

(please check one box) ❒ ❒ ❒

How certain were you of your choice?

| 1 | 2 | 3 | 4 | 5 | 6 | 7 |
| --- | --- | --- | --- | --- | --- | --- |
| Guessing at my choice |  |  | Fairly certain of my choice |  |  | Certain I made the right choice |

**Question 7: Which treatment option do you prefer?**

| **Your health today**  **Usual activities: You have some problems performing usual activities**  **Pain: You are experiencing moderate pain or discomfort**  **Duration of life without treatment: 2 months** |
| --- |

|  | **Treatment A** | **Treatment B** | **Treatment C** |
| --- | --- | --- | --- |
| Your health after treatment | Usual activities:  no problems  Pain: none | Usual activities: some problems  Pain: moderate | Usual activities: some problems  Pain: none |
| Duration of life (years) after treatment | 6 months | 1 year | 10 years |
| One-time Extra tax payment | $450 | $8,100 | $2,700 |

Which treatment do you prefer? Treatment A Treatment B Treatment C

(please check one box) ❒ ❒ ❒

How certain were you of your choice?

| 1 | 2 | 3 | 4 | 5 | 6 | 7 |
| --- | --- | --- | --- | --- | --- | --- |
| Guessing at my choice |  |  | Fairly certain of my choice |  |  | Certain I made the right choice |

**Question 8: Which treatment option do you prefer?**

| **Your health today**  **Usual activities: You have some problems performing usual activities**  **Pain: You are experiencing extreme pain or discomfort**  **Duration of life without treatment: 2 months** |
| --- |

|  | **Treatment A** | **Treatment B** | **Treatment C** |
| --- | --- | --- | --- |
| Your health after treatment | Usual activities: some problems  Pain: none | Usual activities: some problems  Pain: moderate | Usual activities: some problems  Pain: extreme |
| Duration of life (years) after treatment | 6 months | 4 years | 1 year |
| One-time Extra tax payment | $900 | $450 | $2,700 |

Which treatment do you prefer? Treatment A Treatment B Treatment C

(please check one box) ❒ ❒ ❒

How certain were you of your choice?

| 1 | 2 | 3 | 4 | 5 | 6 | 7 |
| --- | --- | --- | --- | --- | --- | --- |
| Guessing at my choice |  |  | Fairly certain of my choice |  |  | Certain I made the right choice |

**Question 9: Which treatment option do you prefer?**

| **Your health today**  **Usual activities: You have some problems performing usual activities**  **Pain: You are experiencing extreme pain or discomfort**  **Duration of life without treatment: 2 months** |
| --- |

|  | **Treatment A** | **Treatment B** | **Treatment C** |
| --- | --- | --- | --- |
| Your health after treatment | Usual activities: some problems  Pain: extreme | Usual activities: some problems  Pain: none | Usual activities: some problems  Pain: moderate |
| Duration of life (years) after treatment | 10 years | 4 year | 6 months |
| One-time Extra tax payment | $450 | $8,100 | $2,700 |

Which treatment do you prefer? Treatment A Treatment B Treatment C

(please check one box) ❒ ❒ ❒

How certain were you of your choice?

| 1 | 2 | 3 | 4 | 5 | 6 | 7 |
| --- | --- | --- | --- | --- | --- | --- |
| Guessing at my choice |  |  | Fairly certain of my choice |  |  | Certain I made the right choice |

**Question 10: Which treatment option do you prefer?**

| **Your health today**  **Usual activities: You have some problems performing usual activities**  **Pain: You are experiencing extreme pain or discomfort**  **Duration of life without treatment: 2 months** |
| --- |

|  | **Treatment A** | **Treatment B** | **Treatment C** |
| --- | --- | --- | --- |
| Your health after treatment | Usual activities: some problems  Pain: none | Usual activities: some problems  Pain: extreme | Usual activities: no problems  Pain: none |
| Duration of life (years) after treatment | 10 years | 4 years | 6 months |
| One-time Extra tax payment | $2,700 | $900 | $450 |

Which treatment do you prefer? Treatment A Treatment B Treatment C

(please check one box) ❒ ❒ ❒

How certain were you of your choice?

| 1 | 2 | 3 | 4 | 5 | 6 | 7 |
| --- | --- | --- | --- | --- | --- | --- |
| Guessing at my choice |  |  | Fairly certain of my choice |  |  | Certain I made the right choice |

**Question 11: Which treatment option do you prefer?**

| **Your health today**  **Usual activities: You have some problems performing usual activities**  **Pain: You are experiencing extreme pain or discomfort**  **Duration of life without treatment: 2 months** |
| --- |

|  | **Treatment A** | **Treatment B** | **Treatment C** |
| --- | --- | --- | --- |
| Your health after treatment | Usual activities: some problems  Pain: extreme | Usual activities:  no problems  Pain: none | Usual activities: some problems  Pain: none |
| Duration of life (years) after treatment | 1 year | 10 years | 6 months |
| One-time Extra tax payment | $2,700 | $8,100 | $900 |

Which treatment do you prefer? Treatment A Treatment B Treatment C

(please check one box) ❒ ❒ ❒

How certain were you of your choice?

| 1 | 2 | 3 | 4 | 5 | 6 | 7 |
| --- | --- | --- | --- | --- | --- | --- |
| Guessing at my choice |  |  | Fairly certain of my choice |  |  | Certain I made the right choice |

**Question 12: Which treatment option do you prefer?**

| **Your health today**  **Usual activities: You have some problems performing usual activities**  **Pain: You are experiencing extreme pain or discomfort**  **Duration of life without treatment: 2 months** |
| --- |

|  | **Treatment A** | **Treatment B** | **Treatment C** |
| --- | --- | --- | --- |
| Your health after treatment | Usual activities:  no problems  Pain: none | Usual activities: some problems  Pain: moderate | Usual activities: some problems  Pain: extreme |
| Duration of life (years) after treatment | 4 years | 1 year | 10 years |
| One-time Extra tax payment | $2,700 | $8,100 | $450 |

Which treatment do you prefer? Treatment A Treatment B Treatment C

(please check one box) ❒ ❒ ❒

How certain were you of your choice?

| 1 | 2 | 3 | 4 | 5 | 6 | 7 |
| --- | --- | --- | --- | --- | --- | --- |
| Guessing at my choice |  |  | Fairly certain of my choice |  |  | Certain I made the right choice |

**Question 13**: Which treatment option do you prefer?

| **Your health today**  **Usual activities: You have some problems performing usual activities**  **Pain: You are experiencing extreme pain or discomfort**  **Duration of life without treatment: 2 months** |
| --- |

|  | **Treatment A** | **Treatment B** | **Treatment C** |
| --- | --- | --- | --- |
| Your health after treatment | Usual activities: some problems  Pain: moderate | Usual activities:  no problems  Pain: none | Usual activities: some problems  Pain: none |
| Duration of life (years) after treatment | 4 years | 10 year | 6 months |
| One-time Extra tax payment | $450 | $8,100 | $900 |

Which treatment do you prefer? Treatment A Treatment B Treatment C

(please check one box) ❒ ❒ ❒

How certain were you of your choice?

| 1 | 2 | 3 | 4 | 5 | 6 | 7 |
| --- | --- | --- | --- | --- | --- | --- |
| Guessing at my choice |  |  | Fairly certain of my choice |  |  | Certain I made the right choice |

**Question 14: Which treatment option do you prefer?**

| **Your health today**  **Usual activities: You have some problems performing usual activities**  **Pain: You are experiencing extreme pain or discomfort**  **Duration of life without treatment: 2 months** |
| --- |

|  | **Treatment A** | **Treatment B** | **Treatment C** |
| --- | --- | --- | --- |
| Your health after treatment | Usual activities:  no problems  Pain: none | Usual activities: some problems  Pain: moderate | Usual activities: some problems  Pain: extreme |
| Duration of life (years) after treatment | 1 year | 6 months | 10 years |
| One-time Extra tax payment | $900 | $2,700 | $450 |

Which treatment do you prefer? Treatment A Treatment B Treatment C

(please check one box) ❒ ❒ ❒

How certain were you of your choice?

| 1 | 2 | 3 | 4 | 5 | 6 | 7 |
| --- | --- | --- | --- | --- | --- | --- |
| Guessing at my choice |  |  | Fairly certain of my choice |  |  | Certain I made the right choice |

**Question 15**: Which treatment option do you prefer?

| **Your health today**  **Usual activities: You have some problems performing usual activities**  **Pain: You are experiencing extreme pain or discomfort**  **Duration of life without treatment: 2 months** |
| --- |

|  | **Treatment A** | **Treatment B** | **Treatment C** |
| --- | --- | --- | --- |
| Your health after treatment | Usual activities: some problems  Pain: moderate | Usual activities: some problems  Pain: none | Usual activities: no problems  Pain: none |
| Duration of life (years) after treatment | 6 months | 4 years | 1 year |
| One-time Extra tax payment | $2,700 | $8,100 | $900 |

Which treatment do you prefer? Treatment A Treatment B Treatment C

(please check one box) ❒ ❒ ❒

How certain were you of your choice?

| 1 | 2 | 3 | 4 | 5 | 6 | 7 |
| --- | --- | --- | --- | --- | --- | --- |
| Guessing at my choice |  |  | Fairly certain of my choice |  |  | Certain I made the right choice |

**Question 16: Which treatment option do you prefer?**

| **Your health today**  **Usual activities: You have some problems performing usual activities**  **Pain: You are experiencing extreme pain or discomfort**  **Duration of life without treatment: 2 months** |
| --- |

|  | **Treatment A** | **Treatment B** | **Treatment C** |
| --- | --- | --- | --- |
| Your health after treatment | Usual activities: some problems  Pain: extreme | Usual activities: some problems  Pain: none | Usual activities: no problems  Pain: none |
| Duration of life (years) after treatment | 4 years | 1 year | 10 years |
| One-time Extra tax payment | $900 | $450 | $8,100 |

Which treatment do you prefer? Treatment A Treatment B Treatment C

(please check one box) ❒ ❒ ❒

How certain were you of your choice?

| 1 | 2 | 3 | 4 | 5 | 6 | 7 |
| --- | --- | --- | --- | --- | --- | --- |
| Guessing at my choice |  |  | Fairly certain of my choice |  |  | Certain I made the right choice |
